# Supplementary material for: Individual and demographic responses of the palm Brahea aculeata to browsing and leaf harvesting in a tropical dry forest of Northwestern Mexico
Source: PeerJ. 2025 Jun 5;13:e19266. doi: 10.7717/peerj.19266 (PMC12145777; doi:10.7717/peerj.19266)
Supplement: Supplemental Information 2 [file peerj-13-19266-s002.docx]

|  | **Leaf production (Leaf ind ind)** | | | | | | | | | | | |
| --- | --- | --- | --- | --- | --- | --- | --- | --- | --- | --- | --- | --- |
| Fixed effects | **Juveniles (10-100 cm)** | | | | **Adults 1 (100.1-250 cm)** | | | | **Adults 2 (>250.1 cm)** | | | |
|  | ***Estimates*** | ***SE*** | ***Z-value*** | ***p-value*** | ***Estimates*** | ***SE*** | ***Z-value*** | ***p-value*** | ***Estimates*** | ***SE*** | ***Z-value*** | ***p-value*** |
| Intercept | 3.9 | 0.06 | 21.6 | <0.001 | 7.3 | 0.05 | 42.3 | <0.001 | 10.6 | 0.07 | 36.2 | <0.001 |
| No-Grazing | 1.1 | 0.08 | 1.3 | 0.20 | 0.9 | 0.05 | 0.7 | 0.49 | 0.9 | 0.06 | 1.8 | 0.07 |
| Harvesting | 1.5 | 0.08 | 6.2 | <0.001 | 1.2 | 0.07 | 2.9 | <0.001 | 1.2 | 0.08 | 1.9 | 0.05 |
| Time (2013) | 1.2 | 0.03 | 7.8 | <0.001 | 1.2 | 0.04 | 4.2 | <0.001 | 1.1 | 0.07 | 1.4 | 0.15 |
| Times (2014) | 1.0 | 0.04 | 0.9 | 0.34 | 1.2 | 0.04 | 5.2 | <0.001 | 1.3 | 0.06 | 3.6 | <0.001 |
| Harv:Time2013 | 0.8 | 0.08 | 2.2 | 0.03 | 1.0 | 0.07 | 0.6 | 0.55 | 1.1 | 0.09 | 0.6 | 0.55 |
| Harv:Time2014 | 0.9 | 0.08 | 1.6 | 0.11 | 0.9 | 0.07 | 1.7 | 0.09 | 0.9 | 0.09 | 0.8 | 0.39 |
| **Random effects** | | | | | | | | | | | | |
|  | SD |  |  |  | SD |  |  |  | SD |  |  |  |
| Individuals | 0.17 |  |  |  | 0.24 |  |  |  | 0.36 |  |  |  |
| Plot | 0.01 |  |  |  | 0.03 |  |  |  | 0.02 |  |  |  |
| Subplot/Plot | 0.01 |  |  |  | 0.001 |  |  |  | 0.02 |  |  |  |
|  |  |  |  |  |  |  |  |  |  |  |  |  |
