# Supplementary material for: Individual and demographic responses of the palm Brahea aculeata to browsing and leaf harvesting in a tropical dry forest of Northwestern Mexico
Source: PeerJ. 2025 Jun 5;13:e19266. doi: 10.7717/peerj.19266 (PMC12145777; doi:10.7717/peerj.19266)
Supplement: Supplemental Information 3 [file peerj-13-19266-s003.docx]

|  | **Leaf length (cm)** | | | | | | | | | | | |
| --- | --- | --- | --- | --- | --- | --- | --- | --- | --- | --- | --- | --- |
| Fixed effects | **Juveniles (10-100 cm)** | | | | **Adults 1 (100.1-250 cm)** | | | | **Adults 2 (>250.1 cm)** | | | |
|  | ***Estimates*** | ***SE*** | ***Z-value*** | ***p-value*** | ***Estimates*** | ***SE*** | ***Z-value*** | ***p-value*** | ***Estimates*** | ***SE*** | ***Z-value*** | ***p-value*** |
| Intercept | 45.24 | 3.53 | 0.79 | 0.89 | 53.1 | 1.8 | 30.2 | <0.001 | 56.48 | 2.54 | 22.27 | **<0.001** |
| No-Grazing | 3.57 | 4.98 | 0.72 | 0.91 | 0.2 | 2.3 | 0.09 | 0.94 | 2.59 | 3.03 | 0.86 | 0.40 |
| **Harvesting** | **5.76** | **1.03** | 5.57 | **<0.001** | 1.5 | 1.4 | 1.0 | 0.3 | 0.33 | 2.28 | 0.15 | 0.89 |
| Time (2013) | 2.40 | 0.38 | 6.26 | **<0.001** | 3.7 | 0.6 | 5.7 | <0.001 | 5.93 | 1.60 | 3.72 | **<0.001** |
| Times (2014) | 2.05 | 0.46 | 4.42 | **<0.001** | 4.1 | 0.7 | 6.1 | >0.001 | 3.64 | 1.54 | 2.36 | **0.02** |
| Harv:Time2013 | -1.94 | 1.10 | -1.77 | 0.08 | 0.7 | 1.1 | 0.6 | 0.53 | -1.39 | 2.29 | -0.61 | 0.55 |
| Harv:Time2014 | -5.25 | 1.05 | -4.98 | **<0.001** | -2.2 | 1.1 | 2.1 | 0.04 | -2.40 | 2.23 | -1.07 | 0.28 |
| **Random effects** | | | | | | | | | | | | |
|  | SD |  |  |  | SD |  |  |  | SD |  |  |  |
| Individuals | 6.07 |  |  |  | 6.5 |  |  |  | 5.57 |  |  |  |
| Plot | 2.20 |  |  |  | 0.001 |  |  |  | 1.31 |  |  |  |
| Subplot/Plot | 11.52 |  |  |  | 2.7 |  |  |  | 1.74 |  |  |  |
